# Dr. Winn on the Occasional Tendency of Iodine to Produce Inflammation of the Joints

**Published:** 1842-08-13

**Authors:** J. M. Winn


*On the occasional tendency of Iodine to produce Inflammation of the Joints.* By J. M. Winn, M. D.—About nine years since the late Dr. Balmanno, of Glasgow, called my attention to a patient of his who had taken iodine under his direction, and whose finger-joints had become swollen during the use of the medicine. Dr. B., at the time, conjectured whether the tumefaction might not have been occasioned by the iodine. As the case was a solitary instance, several gentlemen, who were present, as well as myself felt sceptical as to the fact. I had quite forgotten this circumstance until very lately, when it was recalled to my memory by a somewhat similar case occurring in a young gentleman of Truro, which I saw in consultation with Mr. Jervis of this town. The patient I alluded to was about 20 years of age, and had suffered from hæmoptysis and an indolent superficial inflammation of the fauces; for the latter symptom we repeatedly prescribed, with the best effect, two grains of potas. iodid. three times a day; and the peculiarity of the case was, that as the throat improved in appearance the wrist joint became inflamed and swollen, and on leaving off the iodine the joint speedily recovered its natural state. As this effect occurred every time the iodine was administered, I was led to suspect it was caused by the medicine, and on mentioning my suspicion to Mr. Jervis, he thought he could confirm my opinion by a case of enlarged spleen which he had lately treated successfully by potas. iodid., and in which the joints became swollen on the subsidence of the swelling of the spleen.

From these cases we may conclude that iodine has an occasional tendency to produce inflammation of the joints, and that, when it does produce this effect, it is a sign that the medicine is acting beneficially and effectually on the system. I may incidentally notice that, in the case of the young gentleman alluded to above, the iodine invariably produced, in addition to the inflammation of the joints, an eruption of pustules in different parts of the body; this latter symptom, and also coryza, I have noticed as very common effects of potas. iodid. I would further observe, that the influence which potas. iodid. exerts on the structure of the joints, may be adduced as an additional argument to prove that mercury and iodine have similar effects on the animal economy. Iodine, like mercury, is an effectual remedy for rheumatism, and, like it, we perceive it has also the property of producing a rheumatic sort of inflammation of the tendinous and ligamentous structures.

P. S.—Since writing the above I have met with another case, showing the influence of iodine on the joints. The patient, Mrs. L., of Tywardreath, had been taking potas. iodid. for about a fortnight, under the direction of Mr. Vaudrez, of St. Austell, for an affection of the head, when she complained, with some feelings of alarm, that her finger-joints were swelling. On showing them to me, I attributed the thickening to the action of the medicine. The truth of my opinion was verified by the speedy reduction of the swelling on intermitting the iodine—*Ibid.* July 1, 1842.

[Largely as we have used the iodide of potass, we have never seen such a result as that stated by the author. Indeed, we have found no other inconvenience except occasional fever, headache, flushing of the face, and ill defined feelings of uneasiness at the stomach.]

